# Novel Entropy-Based Phylogenetic Algorithm: A New Approach for Classifying SARS-CoV-2 Variants

**DOI:** 10.3390/e25101463

**Published:** 2023-10-19

**Authors:** Vladimir Perovic, Sanja Glisic, Milena Veljkovic, Slobodan Paessler, Veljko Veljkovic

**Affiliations:** 1 Biomed Protection, Galveston, TX 77550, USAmilena@biomedprotection.com (M.V.); 2 Galveston National Laboratory, Department of Pathology, University of Texas Medical Branch, Galveston, TX 77555, USA; slpaessl@utmb.edu

**Keywords:** entropy, electron–ion interaction potential, COVID-19, phylogenetic analysis, SARS-CoV-2

## Abstract

The SARS-CoV-2 virus, the causative agent of COVID-19, is known for its genetic diversity. Virus variants of concern (VOCs) as well as variants of interest (VOIs) are classified by the World Health Organization (WHO) according to their potential risk to global health. This study seeks to enhance the identification and classification of such variants by developing a novel bioinformatics criterion centered on the virus’s spike protein (SP1), a key player in host cell entry, immune response, and a mutational hotspot. To achieve this, we pioneered a unique phylogenetic algorithm which calculates EIIP-entropy as a distance measure based on the distribution of the electron–ion interaction potential (EIIP) of amino acids in SP1. This method offers a comprehensive, scalable, and rapid approach to analyze large genomic data sets and predict the impact of specific mutations. This innovative approach provides a robust tool for classifying emergent SARS-CoV-2 variants into potential VOCs or VOIs. It could significantly augment surveillance efforts and understanding of variant characteristics, while also offering potential applicability to the analysis and classification of other emerging viral pathogens and enhancing global readiness against emerging and re-emerging viral pathogens.

## 1. Introduction

The COVID-19 pandemic, caused by the novel coronavirus SARS-CoV-2, has dramatically altered the global landscape, leaving no corner of the world untouched. Since its emergence in late 2019, the virus has infected millions of individuals and claimed countless lives, highlighting the urgent need for effective containment strategies. As the scientific community races to understand and combat this relentless virus, monitoring SARS-CoV-2 variants has emerged as a crucial aspect of the ongoing battle.

All viruses, including SARS-CoV-2, change via mutations over time. Most changes have little to no impact on the virus’s properties. However, some changes may affect the virus’s fitness, such as transmissibility, virulence or susceptibility to vaccines and therapeutics. Certain mutations can also negatively impact the detectability of the viral pathogen with conventional diagnostic tests and so derail public health and social measures.

In June 2020, the WHO Virus Evolution Working Group was established with a specific focus on SARS-CoV-2 variants, their phenotypes and their impacts on countermeasures. In late 2020, the emergence of variants that posed an increased risk to global public health prompted WHO to characterize some as “variants of interest” (VOIs) and “variants of concern” (VOCs) in order to prioritize global monitoring and research, required to inform and adjust the COVID-19 response. From May 2021 onwards, WHO began assigning simple, easy-to-say labels for key variants.

Considerable progress has been made in establishing and strengthening a global system to detect signals of potential VOIs or VOCs and rapidly assess the risk posed by SARS-CoV-2 variants to public health. It remains critical that these systems are maintained, and data are shared, according to good principles and in a timely fashion, as SARS-CoV-2 continues to circulate at high levels around the world. While monitoring the circulation of SARS-CoV-2 globally, it also remains essential to monitor their spread in animal populations and chronically infected individuals, which are crucial aspects of the global strategy to reduce the occurrence of mutations that have negative public health implications. In March 2023, WHO updated its tracking system and working definitions for variants of concern and variants of interest [1]. There have been several methods developed for detecting the VOCs and VOIs based on sequences and using mutational entropy [2], evolutionary metrics [3], structural information [4], or machine learning techniques [5].

Phylogenetic analysis is a common tool used for monitoring the evolution of viruses. It involves studying the genetic relationships and evolutionary history of viral strains or variants by analyzing their genetic and protein sequences. Homology-based phylogenetic analysis is a commonly used method to infer evolutionary relationships among organisms based on similarities in their DNA or protein sequences. However, when dealing with highly homologous sequences that differ by only a small number of mutations, this approach can have some weaknesses: (i) the saturation of substitutions which can lead to erroneous inferences about the relationships between sequences, (ii) an insufficient phylogenetic signal to confidently resolve the relationships between closely related sequences, (iii) multiple sequence alignment ambiguities, which can introduce errors in the phylogenetic analysis and lead to incorrect interpretations of the evolutionary relationships, (iv) long branch attraction which can distort the inferred evolutionary relationships, leading to incorrect branching patterns and inaccurate phylogenetic reconstructions, and (v) a lack of independent mutational events, which can make it difficult to resolve the true evolutionary relationships and can lead to ambiguous or conflicting results.

To overcome this challenge and enhance functional sequence analysis, we propose an additional, novel distance measure based on the Informational Spectrum Method (ISM) [6]. The ISM-based phylogenetic approach has been successfully employed in the analysis of various viruses, including influenza [7] and Ebola [8]. Furthermore, we previously utilized the ISM-based phylogenetic approach to evaluate the impact of mutations in the spike protein of SARS-CoV-2 on the efficacy of the COVID-19 vaccine [9]. This approach enables the assessment of the biological implications of mutations, thereby advancing our understanding of viral evolution and vaccine effectiveness.

Here, a novel phylogenetic algorithm utilizing entropy as a protein distance measure has been introduced. The effectiveness of this innovative entropy-based method in discriminating between VOCs and VOIs, surpassing traditional homology-based and ISM-based phylogenetic approaches, has been demonstrated. It has the potential to serve as a valuable tool for monitoring the evolutionary progression of SARS-CoV-2 viruses.

## 2. Materials and Methods

### 2.1. Viruses

We analyzed the subunit 1 of S proteins (SP1) from human SARS-CoV-2 viruses deposited in the GISAID (https://www.epicov.org/epi3/cfrontend#18c7c7, accessed on 13 July 2023). The list of SP1 sequences from analyzed viruses is given in Supplementary Dataset S1.

### 2.2. Protein Sequence Entropy

Protein sequence entropy, or amino acid (AA) based entropy, is defined as the Shannon entropy [10]:

(1) $$SE(X)=\sum_{i=1}^{20}-aa\left(i\right)log(aa\left(i\right))$$

where the aa(i) is the probability of a given amino acid, i.e., the number of the given amino acid in the sequence is divided by N, and N is the length of the protein sequence.

### 2.3. EIIP Entropy

The EIIP entropy is the numerical property of the protein sequence. It is based on the electron–ion interaction potential (EIIP) values of the amino acid sequence (Table 1). The EIIP entropy of the protein sequence X is defined as

(2) $$EE(X)=\sum_{i=1}^{N}-x\left(i\right)log(x\left(i\right))$$

where the x(i) is the EIIP value of the *i*-th amino acid of the sequence X and *N* is the length of the sequence X.

The EIIP is a descriptor of long-range interaction properties, and its definition and properties can be found in previous research [11].

### 2.4. New Protein Distances Based on AA and EIIP Entropies

Let X and Y be two sequences. Then, the AA entropy-based distance between X and Y can be defined as

(3) $$d_{se}(X,Y)=\left|SE\left(X\right)-SE(Y)\right|$$

where the *SE*(*X*) and *SE*(*Y*) are the AA entropy properties of the X and Y sequences, respectively.

In a similar manner, the EIIP entropy-based distance between X and Y can be defined as

(4) $$d_{ee}(X,Y)=\left|EE\left(X\right)-EE(Y)\right|$$

where the *EE*(*X*) and *EE*(*Y*) are the EIIP entropy properties of the X and Y sequences, respectfully.

### 2.5. Algorithm of Generating Entropy-Based Phylogenetic Trees

The AA entropy-based phylogenetic trees can be generated using the following algorithm:

1. For each sequence, calculate its AA based entropy using (1).
2. Calculate the distance matrix with the distance measure defined in (3).
3. Construct the tree using the unweighted pair group method with arithmetic mean (UPGMA) [12] method.

In a similar manner, the EIIP entropy-based phylogenetic trees can be generated using the following algorithm:

1. For each sequence, calculate its EIIP entropy:
  (1) Convert amino acid sequence into signal with EIIP values.
  (2) Calculate EIIP entropy for each sequence using (2).
2. Calculate the distance matrix with the distance measure defined in (4).
3. Construct the tree using the UPGMA method.

### 2.6. Properties of the EIIP Entropy Distance

The EIIP entropy property is not based on multiple sequence alignment (MSA) and does not use any of the substitution model. Therefore, EIIP entropy-based phylogenetic analysis escapes the drawbacks of the MSA-based phylogenetic approaches: insensitivity to a single mutation and position, failure to consider deletion within sequence, time complexity, limited numbers of sequences, ambiguity of the alignment cost criteria, etc. [13].

The EIIP entropy-based distance d is sensitive to a single mutation, the type of the substitution and deletion.

### 2.7. Evolutionary Analyses

The ISM-based phylogenetic tree was generated using (i) the distance measure defined previously in [6], as the absolute difference of the informational spectrum amplitude ratios A(F1)/A(F2), on the characteristic frequencies F1 = 0.257 and F2 = 0.4795, earlier identified in [6], which correspond to the tropism of the H5N1-HPAIV and the seasonal H1N1, respectively, and (ii) using the UPGMA method [12] as the hierarchical clustering method for creation of the ISM based tree.

The traditional homology-based tree was inferred using the UPGMA method [12], where the evolutionary distances were computed using the Poisson correction method [14], and applying the bootstrap test with 500 replicates [15].

All trees were generated using MEGA X software version 10.0.5 [16].

## 3. Results

The World Health Organization (WHO) has suggested the categorization of COVID-19 viruses into “Variants of Concern” (VOC) and “Variants of Interest” (VOI) to enhance surveillance and respond effectively to the dynamic SARS-CoV-2 virus and its effects on public health. Initiatives are underway to devise standardized and universally acknowledged bioinformatics criteria for variant categorization. These efforts comprise the monitoring of viral evolution, the exchange of data and insights, and the refinement of classification criteria as new data emerges. International cooperatives spearheaded by the WHO and other global health institutions aim to standardize variant classification methodologies and establish agreed-upon guidelines. Despite these endeavors, a definitive bioinformatics criterion that distinguishes VOCs from VOIs remains elusive.

**Figure 1:**
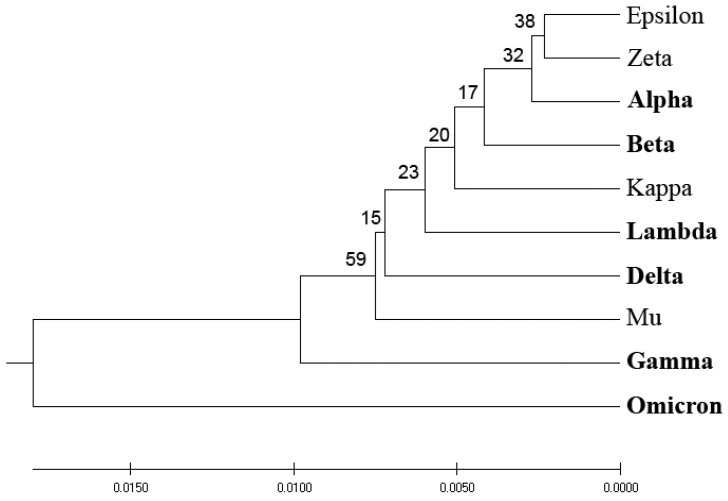
Homology-based phylogenetic analysis of SP1 from VOCs (labeled in bold) and VOIs. The percentage of replicate trees in which the associated taxa are clustered together in the bootstrap test (500 replicates) are shown next to the branches.

We conducted a phylogenetic analysis of the spike protein SP1, a key antigenic component of the SARS-CoV-2 virus, utilizing various protein distance measures. Figure 1 depicts the homology-based phylogenetic analysis of SP1 from VOCs and VOIs. The derived phylogenetic tree shows that VOCs and VOIs are not distinctly separable due to the high homology of these viruses and the small number of mutations distinguishing them.

**Figure 2:**
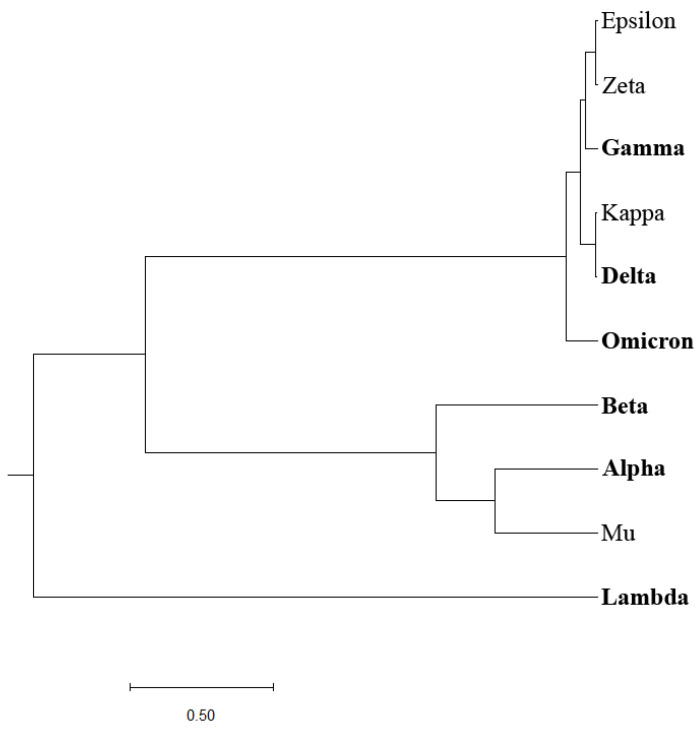
ISM-based phylogenetic analysis of SP1 from VOCs (labeled in bold) and VOIs.

Previously, we designed an Information Spectrum Method (ISM)-based phylogenetic approach that allows the assessment of the biological effects of single mutations and their combinations in proteins [6]. The ISM-based phylogenetic tree is illustrated in Figure 2. Although this approach somewhat better distinguishes VOCs and VOIs compared to the homology-based phylogenetic approach, a clear separation between the two groups of COVID-19 viruses is still lacking.

Entropy calculations for biological macromolecules yield valuable insights into their structural stability, folding processes, ligand binding, thermodynamics, and molecular interactions. Comprehending entropy aids in demystifying the intricacies of these macromolecules and their roles in biological systems. We implemented entropy as a unique distance measure in the phylogenetic analysis of proteins.

**Figure 3:**
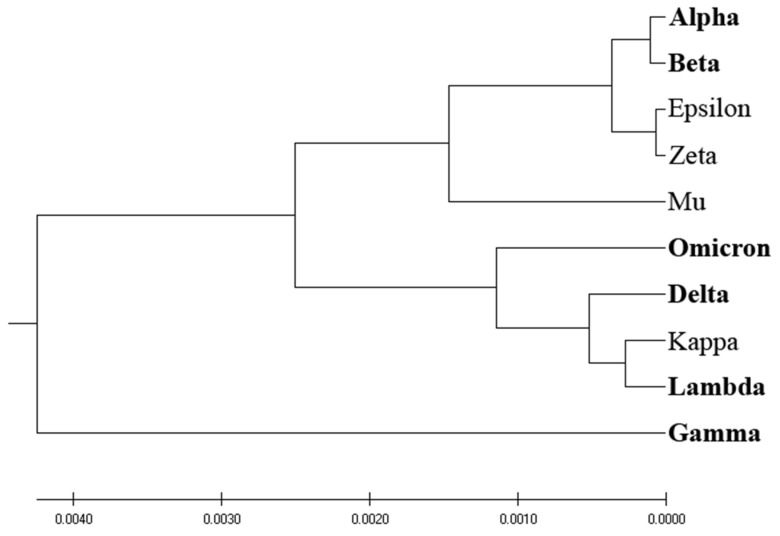
AA-entropy-based phylogenetic analysis of SP1 from VOCs (labeled in bold) and VOIs.

Figure 3 presents the phylogenetic analysis of VOCs and VOIs derived by the entropy calculated by the distribution of amino acids (AA-entropy) in SP1 proteins. However, the segregation between VOCs and VOIs is insubstantial and mirrors that derived by homology-based and ISM-based phylogenetic analyses, as depicted in Figure 1 and Figure 2.

**Figure 4:**
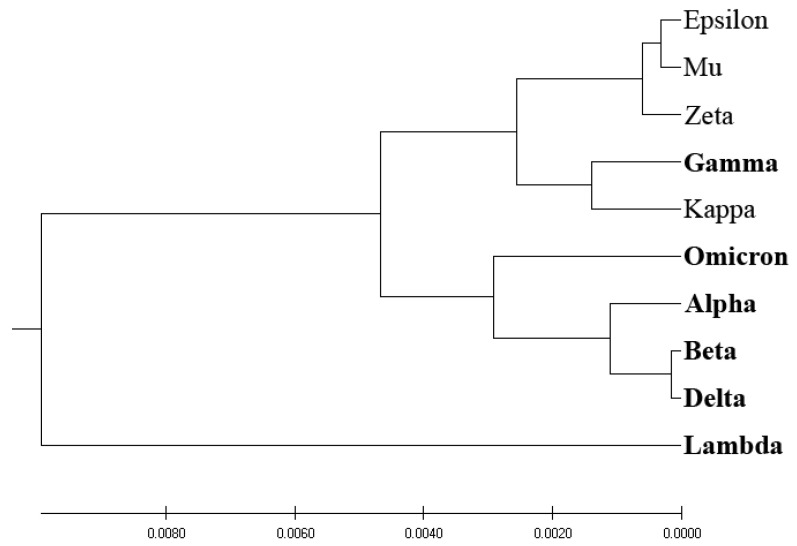
EIIP-entropy-based phylogenetic analysis of SP1 from VOCs (labeled in bold) and VOIs.

Figure 4 showcases the phylogenetic tree obtained via EIIP-entropy as the distance measure, calculated based on the distribution of electron–ion interaction potential (EIIP) values of amino acids in SP1 proteins. Interestingly, the EIIP-entropy-based phylogenetic approach effectively separates VOCs and VOIs, with the exception of the VOC variant Gamma, which is classified with VOIs.

Our analysis reveals that the EIIP-entropy-based phylogenetic approach outperforms the traditional homology-based, ISM-based, and AA-entropy-based phylogenetic methodologies in separating SP1 from VOCs and VOIs. This outcome suggests that employing EIIP-entropy as the distance measure in a novel phylogenetic approach could serve as a valuable tool for classifying emerging SARS-CoV-2 variants as potential VOCs or VOIs.

## 4. Discussion

The WHO introduced the terms VOC and VOI to classify different strains of the SARS-CoV-2 virus, which causes COVID-19 disease. This classification was necessary to distinguish between variants that might pose different levels of risk to global public health. A VOC is a variant of the virus that has shown to be more contagious, more deadly, or more resistant to current treatments and vaccines. The introduction of this term was crucial in guiding public health actions and policies, as well as focusing scientific research on these potentially dangerous strains. A VOI, on the other hand, is a variant that has genetic changes that may affect virus characteristics such as transmissibility and disease severity and may pose a future risk. These are monitored and studied closely to prevent their possible escalation into VOC classification. Classifying new SARS-CoV-2 strains as VOCs or VOIs is essential for monitoring the virus’s evolution, guiding research and public health policies, and prioritizing resources. It helps to understand if a new variant is more transmissible, more dangerous, or resistant to treatments and vaccines. Early identification and understanding of such variants can facilitate appropriate public health actions and interventions to mitigate the spread and impact of the virus.

Developing bioinformatic criteria for discriminating between VOCs and VOIs is crucial for a few key reasons: (i) bioinformatics tools can quickly analyze large volumes of genomic data, allowing for faster detection and classification of new variants; (ii) these tools can provide a more precise and detailed understanding of the genomic changes in new variants, informing their classification; (iii) bioinformatics allow for the analysis of vast amounts of data, crucial given the global scale of the pandemic; and (iv) sophisticated bioinformatics models can potentially predict the impact of specific mutations on viral characteristics like transmissibility and virulence, aiding in identifying potential VOCs or VOIs early. By providing quick, detailed, and scalable analysis, bioinformatics criteria can help streamline the process of variant classification, improving the global response to emerging variants.

As of now, there is not a standardized bioinformatics criterion for distinguishing variants of concern (VOCs) and variants of interest (VOIs) because the impact of specific mutations on virus behavior is not always predictable and often relies on clinical and epidemiological data. While bioinformatics can help identify and analyze genomic changes in new variants, linking these changes to real-world impacts like increased transmissibility, disease severity, or vaccine resistance, this still requires extensive in vitro and in vivo studies and observational data. Therefore, although bioinformatics plays a crucial role in initial identification and surveillance, comprehensive classification requires additional data and analysis.

The spike protein SP1 of the SARS-CoV-2 virus, which causes COVID-19, is a good target for discriminating VOCs and VOIs due to a few key reasons: (i) S1 protein enables the virus to enter host cells. Changes in the spike protein can affect how easily the virus infects cells, which can influence transmissibility and virulence. (ii) S1 protein is the primary target of the immune response, including the response elicited by vaccines. Variations in the SP1can potentially affect vaccine effectiveness. (iii) Many significant mutations identified in VOCs and VOIs so far have occurred in SP1 protein, suggesting it is an area of the virus genome where mutations can have meaningful effects on virus behavior. Monitoring changes in SP1 protein through genomic sequencing can, therefore, provide important clues about how a variant might behave and its potential to become a VOC or VOI.

Here, we developed a novel phylogenetic algorithm for analysis of SP1 proteins from SARS-CoV-2. In this algorithm, as a distance measure for separation proteins, the EIIP-entropy was used. This entropy was calculated based on distribution of EIIP of amino acids in SP1. It was showed (Figure 1, Figure 2, Figure 3 and Figure 4) that the novel EIIP-entropy-based phylogenetic approach outperforms the traditional homology-based, ISM-based, and AA-entropy-based phylogenetic methodologies in separating SP1 from VOCs and VOIs.

In this study, we have pioneered an innovative phylogenetic algorithm for the analysis of SARS-CoV-2’s spike protein SP1. This algorithm leverages the calculation of EIIP-entropy, a measure of distance calculated as the distribution of the EIIP of amino acids within the SP1 protein. A comparative analysis conducted, as visualized in Figure 1, Figure 2, Figure 3 and Figure 4, corroborates that our novel EIIP-entropy-based phylogenetic method exhibits superior performance in distinguishing SP1 proteins from VOCs and VOIs. This comparison was made against traditional homology-based, ISM-based, and AA-entropy-based phylogenetic methodologies.

## 5. Limitations

1. Scope of the dataset: Our research primarily employed the SARS-CoV-2 spike protein as a test case for our ionic entropy-based method. While the results from this singular dataset were promising, we acknowledge that relying on one data point, with a limited number of members might not be representative of the broader potential and applicability of our technique. Testing our method on additional viruses or other molecular scenarios where mutations affect protein folding or activity would provide a more comprehensive view of its effectiveness.
2. Correlation with virulence: A strong validation for our method would indeed involve utilizing a dataset where the virulence of the virus is quantitatively measured. Demonstrating a direct correlation between our entropy measure and the virulence of viruses would strengthen the practical implications of our approach in real-world applications.
3. Comparative assessment: While our ionic entropy method differentiates itself from the traditional Shannon entropy and k-mer based methods, a broader comparative assessment with other proteins beyond the SARS-CoV-2 spike protein is necessary to truly ascertain its advantages and possible drawbacks.

As with all preliminary research, it is vital to understand the limitations of the presented entropy-based method in the domain of protein sequence comparison. We hope that future work will expand upon our foundational study, exploring its applicability and reliability across a wider range of proteins.

## 6. Conclusions

To summarize, the data presented herein strongly suggest that the innovative EIIP-entropy-based phylogenetic approach we have proposed offers a compelling foundation for the development of an instrumental tool for classifying emergent SARS-CoV-2 variants into potential VOCs or VOIs. The algorithm’s potency lies in its enhanced discriminatory capacity for variant classification, which can potentially streamline surveillance efforts and augment our understanding of variant characteristics. Furthermore, given the universality of the principles of protein structure and function, this novel phylogenetic methodology may well hold promise for the analysis and classification of other emerging viral pathogens. This approach’s wider application could potentially enhance our global readiness and response strategies in the face of evolving viral diseases. Moreover, this method could become very useful for rapid detection of mutations leading to “functional changes” of any protein(s) and so enhance our research capabilities in protein research.

## Figures and Tables

**Table 1:** The EIIP used to encode the amino acids.

Amino Acid	EIIP (Ry)
Leu	0.0000
Ile	0.0000
Asn	0.0036
Gly	0.0050
Glu	0.0057
Val	0.0058
Pro	0.0198
His	0.0242
Lys	0.0371
Ala	0.0373
Tyr	0.0516
Trp	0.0548
Gln	0.0761
Met	0.0823
Ser	0.0829
Cys	0.0829
Thr	0.0941
Phe	0.0946
Arg	0.0959
Asp	0.1263

## Data Availability

Data are contained within the article or Supplementary Materials.

## Supplementary Materials

The following supporting information can be downloaded at: https://www.mdpi.com/article/10.3390/e25101463/s1, Dataset S1: The list of analyzed sequences of subunit 1 of S proteins (SP1) from human SARS-CoV-2 viruses deposited in the GISAID (https://www.epicov.org/epi3/cfrontend#18c7c7, accessed on 13 July 2023).
